# Expression of TNF inhibitor gene in the lacrimal gland promotes recovery of tear production and tear stability and reduced immunopathology in rabbits with induced autoimmune dacryoadenitis

**DOI:** 10.1186/1740-2557-2-6

**Published:** 2005-06-28

**Authors:** Melvin D Trousdale, Zenjin Zhu, Douglas Stevenson, Joel E Schechter, Thomas Ritter, Austin K Mircheff

**Affiliations:** 1 Doheny Eye Institute, University of Southern California, Los Angeles, CA; 2 Department of Cell & Neurobiology, Keck School of Medicine, University of Southern California, Los Angeles, CA; 3 Institute of Medical Immunology, Humboldt University, Berlin, Germany; 4 Department of Physiology & Biophysics, Keck School of Medicine, University of Southern California, Los Angeles, CA

## Abstract

**Background:**

The most common cause of ocular morbidity in developed countries is dry eye, many cases of which are due to lacrimal insufficiency. Dry eye affects approximately 10 million in the United States., most of whom are women. In the U.S. alone, an estimated 2 million Sjögren's syndrome patients have dysfunctional lacrimal glands and severe dry eye, and there is no satisfactory treatment. These patients would benefit if their lacrimal tissue function could be restored.

**Methods:**

The effect of adenovirus-mediated transfer of tumor necrosis factor (TNF)-α inhibitor gene on induced autoimmune dacryoadenitis was evaluated in a rabbit model. Soluble transgene protein was detected in tears by ELISA for 7 days following transduction.

**Results:**

Two weeks after induction of disease with activated lymphocytes, tear production, as determined by Schirmer testing, was reduced by about 40%, while tear film stability, as measured by tear breakup time (BUT), declined by 43%. Adenovirus-mediated gene therapy using AdTNFRp55-Ig given 2 weeks after disease induction, resulted in the return of tear production to normal levels by week 4. In the treated disease group, tear BUT improved significantly by week 4. Rose bengal scores, an indicator of corneal surface defects, increased after disease induction and declined after gene therapy. In the lacrimal gland, the CD4 to CD8 T cell ratio was 4:1 in the disease group compared to 1:2 in the treated group. Infiltration of T cells and CD18+ cells was reduced approximately 50% after gene therapy.

**Conclusion:**

We concluded that therapeutic levels of soluble TNF inhibitor were achieved in the lacrimal gland and on the corneal surface. Anti-inflammatory cytokine gene expression might offer a potential therapeutic modality for the treatment of autoimmune dacryoadenitis, once suitable vectors become available.

## Background

Dry eye affects approximately 10 million Americans, most of who are women. One of the most severe forms of dry eye is found in Sjögren's syndrome patients. A combination of immunologic, genetic, hormonal, and environmental factors may play a role in the development of autoimmunity involving the lacrimal gland [1-3]. Lacrimal insufficiency is responsible for some severe forms of dry eye and may be caused in part by lymphocytic infiltration. The inflammatory infiltrates produce toxic factors that act as immune mediators, resulting in reduced secretory function caused by secretory tissue atrophy and dysfunction of the surviving tissue [4,5].

Both murine and rabbit models have been used to elucidate the mechanisms of autoimmune lacrimal gland disease. Our Ocular Surface Center research group reported that autologous peripheral blood lymphocytes (PBL) proliferate when co-cultured with rabbit lacrimal gland epithelial cells [6]. These activated lymphocytes induced dacryoadenitis within 2 weeks when injected into the donor rabbit's remaining contralateral gland, providing an in vivo model of autoimmune dacryoadenitis [7,8]. The induced lacrimal gland dysfunction, characterized by reduced basal tear production, reduced tear stability, abnormal corneal surface staining, and increased presence of CD4+ T cells, mimics several important features of keratoconjunctivitis sicca and Sjögren's syndrome.

Tumor necrosis factor (TNF)-α is a pro-inflammatory cytokine produced mainly by monocytes and macrophages, although numerous other cells are also producers. It is a pleiotrophic cytokine with two known receptors and many regulatory effects, including cytotoxicity, anti-infection, growth modulation, and cellular differentiation. Kolls *et al*. [9] produced a recombinant protein capable of binding and neutralizing TNF and lymphotoxin. The product, a fusion protein formed by joining the human 55-kDa TNF receptor extracellular domain to a mouse IgG heavy chain, binds TNF by engaging two of its three receptor sites. Kolls *et al*. [9] constructed an adenoviral vector with a cytomegalovirus promoter encoding the chimeric TNF inhibitor (AdTNFRIp55-Ig). Later, in a transplantation model, Ritter *et al*. (2000) [10] demonstrated that the vector might be a useful gene therapy tool for controlling inflammation. Using the same vector, we demonstrated that expression of the TNF-inhibitor gene suppressed activation of lymphocytes by lacrimal epithelial cells in mixed cell reactions [11].

Previously, in a prophylactic study utilizing the same clinical parameters, we reported evidence that soluble TNF-inhibitor protein partially suppresses the appearance of Sjögren's syndrome-like features of reduced basal tear production, as well as the immunohistopathology associated with induced autoimmune dacryoadenitis [12]. The current manuscript presents evidence that AdTNFRIp55-Ig gene therapy treatment of rabbits with established autoimmune dacryoadenitis results in improvement of clinical features, such as an increase in basal tear production, an increase in tear stability, and a reduction in surface corneal defects. The therapy also reduced the intensity of immune cell infiltration.

## Methods

### Animals, surgical procedures and clinical assessments

Female New Zealand white rabbits (3.5–4 kg) were obtained from Irish Farms (Norco, CA). All animals were used in accordance with the Association for Research in Vision and Ophthalmology (ARVO) Resolution on Use of Animals in Ophthalmic Research. Animals were maintained in a facility fully accredited by the American Association for Laboratory Animal Science. Clinical tests were performed on all eyes prior to experimentation. The eyes were initially examined by two investigators using slit-lamp biomicroscopy, Schirmer testing after topical anesthetic eye drops (0.5% tetracaine), determination of tear film breakup time (tear BUT), and rose bengal staining, and no corneal defects were detected. Schirmer test paper strips for measuring tear production were purchased from Chauvin Pharmaceuticals Ltd (Romford, Essex, U.K.), fluorescein from Alcon Laboratories Inc, (Fort Worth, TX.) and rose bengal strips from Akorn, Inc., (Abita Springs, LA).

Results were recorded on a cornea diagram and scored using a standardized grading system [13]. The intensity of staining of the medial and lateral bulbar conjunctiva and the cornea was graded; the maximum grade for each of these three tests was 3, for a total maximum grade of 9 per eye.

After anesthesia, the left lacrimal gland was surgically removed from each rabbit for preparation of the purified lacrimal gland epithelial cells (pLGEC) as previously described [6,11]. Peripheral blood was also taken for lymphocyte preparation.

### Cell culture and immunocytochemical reagents

Hepato Stim Culture Medium was purchased from Becton Dickinson, (Bedford, MA). Ham's F12 medium and antibiotic/antimycotic mixtures were purchased from Gibco BRL products, (Rockville, MD). Dulbecco's modified Eagle's medium and fetal bovine serums were purchased from Omega Scientific, (Tarzana, CA). Bovine serum albumin (BSA), soybean trypsin inhibitor, and linoleic acid were purchased from Sigma Chemical Co., (St. Louis, MO). Tritiated thymidine (^3^H-thymidine) was purchased from DuPont NEN Research Products, (Wilmington, DE). Monoclonal antibodies to rabbit CD4, CD8, and CD18 were purchased from Spring Valley Labs, (Woodbine, MD). Goat antiserum to rabbit T lymphocyte antigen (RTLA) was obtained from Cedar Lane Laboratories (Hornby, Ontario, Canada). Biotin-labeled goat anti-mouse IgG Fc and donkey anti-goat IgG were purchased from Chemicon International, (Temecula, CA). The Vectastain Elite ABC kit was obtained from Vector Laboratories Inc., (Burlingame, CA). Propidium iodide was purchased from Sigma (St. Louis, MO).

### Autologous mixed cell reaction and induction of autoimmune dacryoadenitis

Descriptions of lacrimal gland excision, acinar cell purification, and mixed cell reaction procedures have been published previously [6,7,12]. Purified lacrimal gland epithelial cells and PBL were isolated and cultured separately for 2 days. Mixed cell reactions were performed using equal numbers (1 × 10^6^) of autologous PBL and 2500 RAD γ-irradiated pLGEC, then co-cultured for 5 days. The cells were also cultured in 96-well plates under the same conditions, but using 1 × 10^5 ^cells of each type to monitor PBL proliferation. The cells were incubated with 1 μ Curie of ^3^H-thymidine and harvested 24 hr later using a Brandel model 290 PHD™ sample harvester (Gaithersburg, MD). An LS 6000IC beta scintillation counter (Beckman Instruments, Inc., Fullerton, CA) was used to measure ^3^H-thymidine incorporation. A minimum of 6 wells was counted to obtain representative data. Peripheral blood lymphocytes from mixed cell reactions with a stimulation index greater than 2 were considered to contain activated lymphocytes and, therefore, to be suitable for the induction of dacryoadenitis.

Two million stimulated lymphocytes were injected into the central region of the inferior lacrimal glands of the right eyes of the respective donor rabbits using a 25-gauge butterfly needle on a tuberculin syringe. To avoid tissue damage caused by rapid volume expansion, the micro-volume delivery system was set to deliver 10 μl at 10-sec intervals (10 μL × 20 repetitions = 200 μL). Rabbits receiving stimulated cells from the mixed cell reaction are hereafter referred to as induced dacryoadenitis (ID) animals.

### Vectors and in vivo transduction

An adenovirus construct carrying the TNF-inhibitor gene (AdTNFRp55-Ig) described previously was used [9,10]. Briefly, a fusion protein was formed by joining the human 55-kDa TNF receptor (TNFRp55) extracellular domain with a mouse IgG heavy chain (TNFRp55-Ig) for stability. It was then subcloned into the pACCMV vector to generate recombinant Ad expressing the TNFRp55-Ig chimeric molecule. This Ad vector, which had a promoter from the human cytomegalovirus, expressed soluble TNFRp55-Ig that had the ability to completely block TNF-α and lymphotoxin-α. Half of the ID animals described above were transduced with AdTNFRp55-Ig (1 × 10^8 ^pfu in 0.2 ml injected into each lacrimal gland after clinical symptoms showed established disease. These animals are hereafter referred to as ID/treated or ID/AdTNFRI). There were 5 or more animals in each study group.

### Tissue collection and immunohistopathology

All animals were sacrificed at the end of 4 weeks (i.e. week 4 was 2 weeks after gene therapy). Inferior lacrimal glands of the right eyes were excised and dissected longitudinally into two parts. One part was fixed in 10% formalin and embedded in paraffin; the second part was embedded in optimal cutting temperature (OCT) compound (Miles, Inc., Elkhart, NJ), then snap-frozen in liquid nitrogen and stored at -70°C until histologic examination. Cryosections of the transduced tissues were fixed in 4% paraformaldehyde, then counterstained with propidium iodide (50 μg/ml). A minimum of 5 fields were randomly selected and photographed with a confocal microscope for cell analysis. The paraffin-embedded tissue samples were sectioned at 5 μm, then deparaffinized and stained with hematoxylin and eosin (H&E) for light microscopic examination. The OCT-embedded tissue samples were also sectioned at 5 μm using a cryostat, fixed in chilled acetone (4°C), air-dried, then rehydrated in phosphate-buffered saline for immunohistologic staining. After the sections were blocked with 5% BSA for 15 min, they were incubated either at room temperature for 1 hr or in a humidified chamber at 4°C overnight with primary antibodies: mouse anti-CD18 (1:1000), goat anti-RTLA (1:300), CD4, or CD8 (1:200). The sections were then incubated with biotinylated species-specific secondary antibody for 60 min at room temperature. The sections were rinsed again, then quenched with 0.3% H_2_O_2 _in 40% methanol for 15 min, incubated in ABC reagent for 30 min, rinsed three more times, then developed for 3 min. Negative controls were performed for each antibody using 1% normal sera from the same species that the primary or secondary antibodies were derived from as substitutes for either primary or secondary incubation. Hematoxylin counterstain was applied before mounting. Cells that stained positive exhibited an intense brown color and were readily distinguished from the blue background. The entire section was scanned and analyzed with an Automated Cellular Imaging System (ACIS^®^: ChromaVision Medical Systems, Inc., San Juan Capistrano, CA). ACIS, a proprietary, color-based imaging technology with automated microscopy, provides quantitative data, including percent positive, intensity scoring, and area measurement. ChromaVision system is designed to measure the total area occupied by all cells in a scanned section and the percentage of that area that is occupied by cells expressing a specific cell marker for CD4, CD8, CD18, or RTLA. Four sections from each gland were scanned for a total of 20 scanned sections per group. Data are shown as mean positive percentage ± SD.

### Statistics

Data collected from clinical analysis and ChromaVision were subjected to paired *t*-test or signed rank sum test and analysis of variance (ANOVA). Multiple comparison *t*-tests were calculated when ANOVA *p ≤ *0.05. Statistical significance was adjusted for the number of pairwise comparisons with a Bonferroni correction.

## Results

### Induced autoimmune dacryoadenitis and clinical assessment

**Figure 1:**
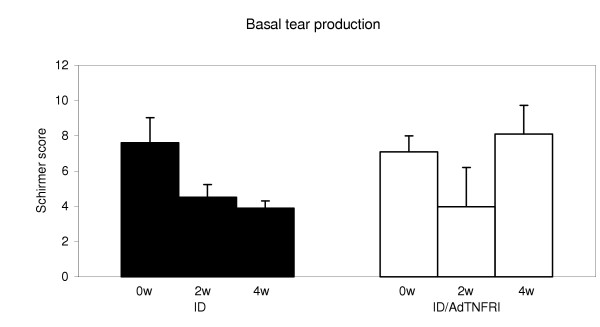
**Basal tear production was measured by Schirmer test in 10 rabbits (0 w)**. Dacryoadenitis was induced in these rabbits using lymphocytes activated in a mixed cell reaction. These rabbits were designated the induced dacryoadenitis (ID) group. At 2 w and 4 w, tear production in the ID group was significantly reduced (p = 0.005 and 0.006, respectively) compared to tear production at 0 w. The Schirmer test was repeated at 2 w for those animals to be treated; tear production was significantly reduced (p = 0.04) compared to at 0 w. At 2 w, 5 of the 10 ID animals received an injection of 1 × 10^8 ^pfu of AdTNFRp55-Ig into the remaining lacrimal gland. These animals were designated the ID/AdTNFRI treatment group. At 4 w (2 w after treatment) tear production was remeasured. There was no significant difference in tear production in the treatment group at 4 w compared to at 2 w (p = 0.52); however, there was a significant difference in tear production between the treated and untreated groups at 4 w (p = 0.04).

**Figure 2:**
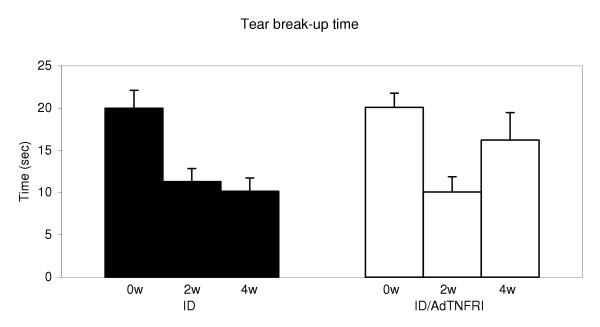
**Tear break-up time (BUT) was measured at 0 w, 2 w, and 4 w. **In the ID group, tear BUT was significantly decreased at 2 w and 4 w compared to at 0 w (within group comparison p = 0.0002). Tear BUT was significantly different for the ID/AdTNFRI group (p = 0.002) at 2 w, before treatment, but not at 4 w, after treatment (p = 0.09). A between groups comparison (ID and ID/AdTNFRI) revealed a significant difference only at 4 w (p = 0.005).

**Figure 3:**
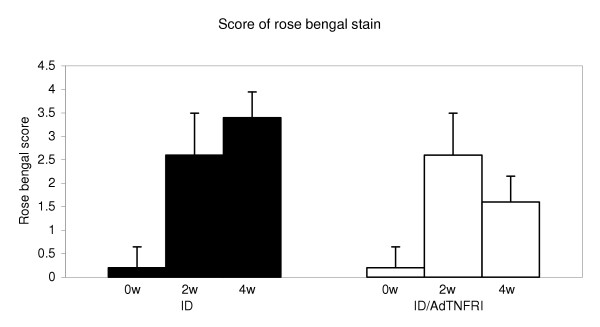
**Deficiency in preocular tear film protection was determined with rose bengal stain; the ID group was compared to the ID/AdTNFRI group. **Staining at 2 w and 4 w was significantly different from at 0 w (p = 0.001 and 0.002, respectively) within the ID group, as well as within the ID/AdTNFRI group (p = 0.001 and 0.006, respectively). A between groups comparison (ID versus ID/AdTNFRI) revealed a significant difference only at 4 w (p = 0.0008).

Autologous PBL, activated by co-culture with rabbit lacrimal gland epithelial cells, induced dacryoadenitis within 2 weeks when injected into the donor rabbit's remaining contralateral lacrimal gland. Clinical assessments, including basal tear production (Fig. 1), tear breakup time (Fig. 2) and rose bengal staining (Fig. 3) were performed on 10 rabbits at 0, 2 and 4 weeks after induction of disease. Before disease induction, the Schirmer test scores based on 1 min readings, representing normal basal tear production for the two study groups were 7.6 to 7.1 mm (Fig. 1). Two weeks after disease induction, tear production dropped more than 40% to scores of 4.5 and 4.0, respectively. The lacrimal glands of animals in one group were then transduced with AdTNFRp55-Ig; clinical assessment tests were repeated 2 weeks later. Schirmer scores for the gene therapy group (ID/treated) increased to 8.1 mm, indicating improved tear secretion, while Schirmer scores for the untreated disease group (ID) decreased to 3.9 mm.

Before disease induction, tear BUT was 20 sec. Two weeks after disease induction, tear BUT decreased to11.3 and 10.1 sec, indicating a loss of tear stability for both groups. Four weeks after disease induction, tear BUT for the treated group was closer to normal (16.2 sec) while in the untreated group, tear stability remained unchanged (10.2 sec).

Rose bengal staining scores before disease induction started at 0.2 and increased to 2.6 for both groups at 2 weeks. At 4 weeks, scores for the treated group decreased to 1.6 while scores for the untreated group continued to increase (3.4).

### Immune cell infiltrates

**Figure 4:**
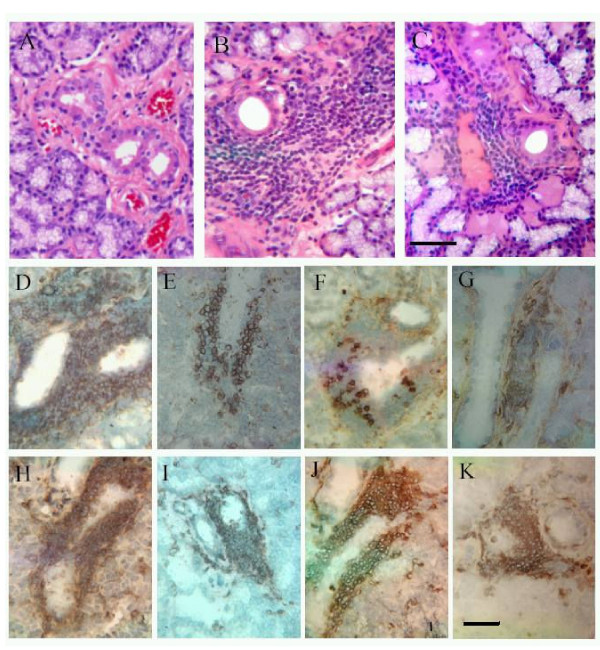
**Hematoxylin & eosin stained lacrimal sections from animals designated normal (A), ID (B), and ID/AdTNFRI (C). **(Bar, 40 μm). Immunohistochemical staining of ID and the diseased treated group ID/AdTNFRI cryosections (Bar, 20 μm) for CD4 (D) and (E), respectively; for CD8 (F) and (G), respectively; for RTLA (H) and (I), respectively; for CD18 (J) and (K), respectively.

Microscopic examination of H&E-stained normal lacrimal gland (Fig. 4A) revealed sparsely scattered lymphocytes, plasma cells, and macrophages located throughout the gland. Glands from animals with induced dacryoadenitis (without and with treatment in Fig. 4B and 4C, respectively) contained immune cell infiltrates, consisting of small to large foci that stained positive for CD4 (Fig. 4D and 4E, respectively), CD8 (Fig. 4F and 4G, respectively), RTLA (Fig 4H and 4I, respectively), and CD18 (Fig 4J and 4K, respectively) antigens located between acini and around ducts and venules. Occasionally, CD8+ cells were seen within acinar and ductal epithelium, and some areas had atypical acinar morphology.

Table 1 provides a summary of the quantitative analysis of CD4+, CD8+, RTLA+, and CD18+ immune cells present in lacrimal gland tissue. In normal tissue, the ratio of CD4+ to CD8+ stained area was approximately 1:1.4 (0.34% and 0.49% of the stained area was occupied by CD4+ and CD8+ T cells, respectively). Four weeks after disease induction, the ratio changed to about 3:1 (i.e. 1.20% and 0.47% as explained above). In the normal lacrimal gland sections, 1.65% of the area was RTLA+ and 0.17% was CD18+. Four weeks after disease induction, there was a significant increase of RTLA+ and CD18+ cells in the lacrimal tissue (13.2% for RTLA+ and 9.68% for CD18+ cells). After gene therapy, the numbers of CD4+, RTLA+ and CD18+ cells declined, and the CD4:CD8 ratio reverted to 1:2 (0.8% and 1.63%, respectively). Compared to ID group, significant decrease of CD4+, RTLA+ and CD18+ cells was found in AdTNFRI treated lacrimal glands (p < 0.001), whereas CD8+ cells increased significantly following gene therapy (p < 0.0001).

**Table 1:** Statistical analysis for Therapeutic TNF-inhibitor gene therapy study:

	**Normal**	**ID**	**ID+AdTNFRI**
			
**CD4**	0.34 ± 0.09^1^	1.20 ± 0.45	0.80 ± 0.34
Anova p < 0.0001			
Normal		p < 0.0001	p = 0.008
ID			p = 0.0003
			
**CD8**	0.49 ± 0.08	0.47 ± 0.14	1.63 ± 0.94
Anova p < 0.0001			
Normal		p = 1.00	p = 0.0002
ID			p < 0.0001
			
**RTLA**	1.65 ± 0.84	13.2 ± 4.29	7.17 ± 2.81
Anova p < 0.0001			
Normal		p < 0.0001	p = 0.0001
ID			p < 0.0001
			
**CD18**	0.17 ± 0.08	9.68 ± 2.55	5.53 ± 2.32
Anova p < 0.0001			
Normal		p < 0.0001	p < 0.0001
ID			p < 0.0001

^1 ^Mean ± SD

^2 ^Pairwise comparison p-value with a Bonferroni correction.

ID = induced dacryoadenitis; ANOVA = analysis of variance.

Compared to ID group, AdTNFRI treated lacrimal glands have a significant decrease of CD4+ (p = 0.0003), RTLA+ (p < 0.0001) and CD18+ cells (p < 0.0001), and a significant increase of CD8+ cells in these treated glands (p < 0.0001).

## Discussion

The lacrimal glands produce most of the fluid that is essential for keeping the ocular surface tissue healthy and for maintaining a smooth refractive interface for light entering the eye. Lacrimal insufficiency can result from both immune and nonimmune mechanisms. In our animal model of autoimmune dacryoadenitis, activated autologous lymphocytes trigger a lymphocytic infiltration that impairs glandular function, mimicking Sjögren's syndrome. In this system, the lacrimal gland epithelial cells become dysfunctional, resulting in reduced tear production and a loss of tear stability [12].

In a previous *in vitro *gene transfer study, we reported transducing cultured lacrimal gland epithelial cells with adenovectors carrying a TNF-inhibitor gene, TNFRp55-Ig [11]. Expression of the transgene in these cells suppressed their ability to activate lymphocytes in a mixed cell reaction. The soluble TNF-inhibitor gene product was present in the culture media of the transduced cells for up to 4 weeks. In a later study involving the *in vivo *transduction of the lacrimal gland with the same vector, the soluble TNF receptor transgene protein was detectable in tears after 7 days, but not at 14 days [8]. This transient expression is a well-known feature of adenovirus-mediated gene transfer and is thought to be a consequence of nonintegration of the transgene DNA into the cell genome and possibly the elimination of transduced cells by the immune system. In spite of this transient gene expression, there was a beneficial impact on the induced disease. Basal tear production, tear stability, and ocular surface defects, three common clinical parameters that are used to diagnose dry eye [1], were improved. Without treatment, tear production and tear BUT declined by about 45% to 50%, and ocular surface damage increased by 60%, based on a scoring system for rose bengal staining that has high sensitivity and specificity for surface defects. The intensity of immune cell infiltration in the lacrimal gland also declined.

Adenovirus vectors used in gene therapy can elicit an immune response in the recipient. Previously, we reported that our adenovirus vector, expressing the green fluorescent protein (GFP) gene using the same CMV promoter as in AdTNFRp55-Ig, induced similar numbers of CD4+ and CD8+ T cells to what is found in normal nontransduced glands, although numbers of RTLA+ and CD18+ cells present in lacrimal tissue did increase following transduction [8].

We also reported that reduced tear production and tear break-up time was detectable 2 weeks after induction of dry eye disease and that these clinical features persisted for at least 6 more weeks on our model [12]. For this current study, we elected to intervene therapeutically after acute disease was recognizable (i.e. 2 weeks after initiating disease) and to evaluate short-term gene therapy treatment at 4 weeks knowing that adenovirus-mediated TNF-inhibitor transgene expression would be less than 2 weeks in our system [8]. In the current studies we demonstrated that even transient expression of the TNF-inhibitor gene in the lacrimal tissue has a desirable impact on clinical and pathological features of established autoimmune dacryoadenitis. For future studies, it would seem that a long-term transgene expression would be appropriate; therefore our follow-up study will test long-term expression using an AAV vector carrying the TNF-inhibitor gene.

## Conclusion

Adenovirus-mediated transfer of the TNFRp55-Ig gene to the lacrimal gland resulted in expression of the transgene product and its secretion into tears. The presence of the TNF inhibitor protein in the lacrimal gland promoted recovery of tear production and tear stability and reduced immunopathology in rabbits with established autoimmune dacryoadenitis. It appears that severe dry eye may benefit by down regulating TNF.

## Competing interests

The author(s) declare that they have no competing interests.

## Authors' contributions

MDT and DS designed the studies, helped with the interpretation and the writing of the manuscript. ZZ was primarily involved in carrying out the clinical assessments and the acquisition of immunocytochemistry data. JES and AKM helped plan the protocol and interpret the data for important intellectual content. TR was involved with planning all aspects involving the gene therapy and interpretation of the results.
